# Multi-Angle Crack Detection in CFRP Based on Line Laser Infrared Thermography Scanning Technology

**DOI:** 10.3390/polym17040508

**Published:** 2025-02-15

**Authors:** Guangyu Zhou, Zhijie Zhang, Wuliang Yin, Yu Fu, Ding’erkai Wang

**Affiliations:** 1 School of Instrument and Electronics, North University of China, Taiyuan 030051, China; zgy4083273@163.com (G.Z.); wangdek0903@163.com (D.W.); 2 School of Electrical and Electronic Engineering, The University of Manchester, Manchester M13 9PL, UK; wuliang.yin@manchester.ac.uk; 3 Institute of Technology, Shanxi Open University, Taiyuan 030027, China; 15735112962@163.com

**Keywords:** CFRP, multi-angle cracking, infrared thermography, line laser scanning

## Abstract

Infrared thermography is a real-time and efficient method for defect detection. This study utilizes line laser scanning infrared thermography to detect cracks in manually laid-up unidirectional CFRP, 3D-printed CFRP cracks, and naturally occurring microcracks in CFRP deflectors. In manually layered unidirectional CFRP, detection performance is influenced by the layup direction, with cracks aligned to the layup exhibiting minimal hindrance to heat conduction, resulting in weaker high-frequency components in thermal images and poorer detection accuracy. In contrast, the composite structure of 3D-printed CFRP minimizes the impact of crack orientation. By analyzing the temperature characteristics of the crack center and thermal drag tail for cracks with varying opening angles, the study establishes a relationship between the crack opening angle, crack depth, and thermal features. Fitted curves of the ratio between crack opening angle and absolute temperature difference yielded an average *R*^2^ of 0.9828 and MSE of 0.1287, validating the effectiveness of the proposed approach. Finally, the features of microcracks in CFRP deflector plates were effectively extracted through high-frequency filtering, which demonstrated the broad applicability and robustness of this study.

## 1. Introduction

Carbon fiber-reinforced composites (CFRPs) offer numerous advantages, including high strength, a low thermal expansion coefficient, and excellent heat resistance, making them widely used in aerospace, rail transportation, and other industries. Common manufacturing methods for CFRP materials include manual lay-up, prepreg lay-up, and thermo-compression molding. In recent years, 3D printing technology has also been employed for CFRP fabrication. This method builds components layer by layer, enabling the creation of complex, integrated geometric structures and effectively addressing the issue of delamination commonly observed in conventionally bonded structures. However, during the 3D printing process, defects such as scratches, cracks, and deformations can arise due to issues like improper temperature control and inconsistent material extrusion rates, significantly compromising the performance of the final product. To address these challenges, researchers have utilized various inspection techniques for CFRP components, including infrared thermography [1], ultrasonic testing [2], X-ray inspection [3], and acoustic emission testing [4], all of which play critical roles in the quality assurance of composite materials.

Infrared thermography is a relatively recent method of non-destructive testing that has gained widespread use due to its advantages, including non-contact operation, visualization, high efficiency, and the ability to perform quantitative analysis [5]. In active infrared thermography, an excitation source is used to thermally stimulate the object under investigation. Various excitation sources, such as lasers [6], halogen lamps [7], and ultrasonic waves [8], can be employed to provide the necessary thermal energy for the test. AbouelNour et al. investigated the detection effectiveness of embedding different numbers of defects in 3D-printed specimens using optical infrared thermography [9]. Lee et al. examined defect detection in 3D-printed carbon fiber-reinforced composite cylindrical structures utilizing laser ultrasound thermography [10]. Optical thermography-based methods can effectively detect defects in 3D-printed and molded CFRP specimens; however, detecting tiny cracks remains challenging. The advantage of lasers lies in their ability to transmit heat over long distances, and by combining optical lenses, the laser beam can be shaped into various forms. This shaped laser beam can then be scanned to achieve large-area, high-efficiency defect detection. Montinaro et al. used a point laser to scan the surface of the material, capturing the thermal field generated by the moving heat source with an infrared camera to detect debonding defects on laminates. This method successfully identified the location and size of the defects [11]. However, the coverage area of a point laser is limited, resulting in low detection efficiency. To address this, researchers often combine lenses to create a laser array or linear light source. Wei et al. proposed Laser Arrays Scanning Thermography (LAsST), which employs a multipoint laser array to form a surface light source and achieves high-efficiency detection through scanning. However, laser arrays generate higher power consumption [12]. Liu et al. introduced Line Laser Fast Scanning Thermography (LLFST) for detecting artificial debonding defects in thermal barrier coatings and discussed the trailing phenomenon caused by line laser fast scanning. However, it does not suggest a better way to remove the trailing phenomenon [13]. Li et al. proposed a correction method for thermal sequence image aberration during continuous line laser inspection and explored its applicability at different scanning speeds. However, the inspection primarily targets large-sized defects, making it challenging to accurately detect and characterize small defects [14]. Ahmadi et al. used the point-by-point laser scanning method for detecting cracks in 3D-printed metals, achieving higher resolution characterization through a super-resolution approach combined with conventional thermographic reconstruction techniques [15]. Hwang et al. employed a continuous scanning line laser thermography system for wind turbine blade inspections and introduced a coordinate transformation method for spatio-temporal integration, converting moving scans into fixed-viewpoint inspections to visualize hidden layered defects [16]. Puthiyaveettil proposed a line laser scanning thermography moving inspection model for surface crack detection and successfully detected steel cracks at high temperatures of 600 °C [17]. Pech-May utilized continuous line lasers for semi-automated detection of cracks in rails and gears with complex shapes, mapping the crack shapes and locations in a 3D model of the specimen [18].

After acquiring infrared images, it is often necessary to characterize the shape of defects through a series of processing methods. Classical algorithms, such as wavelet transform, principal component analysis (PCA) [19], and fuzzy clustering algorithms [20], are commonly employed for infrared image denoising, enhancement, and defect edge characterization. However, many studies do not analyze the detection results of cracks from multiple angles. In this study, cracks on the surface of manually layered CFRP as well as CFRP made by 3D printing were detected using line laser scanning thermography. In the case of manually layered CFRP, cracks in multiple directions were introduced to explore the detection effectiveness of line laser scanning for cracks oriented in different directions. The imaging effect of cracks in directions different from the layup was examined using a high-frequency filtering enhancement method. In 3D-printed CFRP, the issue of detection discrepancies due to different layup directions was avoided. The thermal imaging characteristics of cracks with various opening angles were investigated, and the curves representing the ratio of crack opening angle to temperature difference were fitted by calculating the ratio of the crack center temperature to the crack thermal drag tail. The relationship between crack opening angle, crack depth, and temperature characteristics was analyzed and summarized. Since CFRP cracks in practical applications do not appear in a single direction, investigating multi-directional cracks with varying opening angles enables classification of their thermal imaging characteristics. This, in turn, helps identify the most effective detection strategies for different crack conditions. Finally, naturally occurring microcracks (scratches) in the CFRP deflector specimens were detected, and their features, which are nearly invisible in low light, were effectively extracted through the high-frequency filtering of the differential images. This process verified the universality of the study.

## 2. Theoretical Foundation

### 2.1. Line Laser Scanning for Heat Transfer Principle

For a homogeneous, isotropic material, the three-dimensional heat transfer equation can be expressed as:

(1) $$\rho c_{P}\frac{\partial T}{\partial t}=\nabla \cdot \left(k\nabla T\right)+Q$$

where *ρ* is the density of the material in kg/m^3^, *c_P_* is the specific heat capacity of the material in J/(kg·°C), *Q* is the internal heat source term in W/m^3^, and *k* is the thermal conductivity of the material in W/(m·°C), which characterizes the material’s ability to conduct heat. ∇*T* is the temperature gradient in °C/m.

As the line laser moves across the surface of the sample, the resulting heat source can be expressed in the form of a Gaussian distribution:

(2) $$Q\left(x,y,t\right)=\frac{P}{\pi r^{2}}\exp\left(\frac{{\left(x-vt\right)}^{2}+y2}{r^{2}}\right)$$

where *P* is the laser power, *r* is the half-width of the laser spot. *v* is the laser scanning speed, (*x*, *y*) are the spatial coordinates, and *t* is the time.

During the inspection process, when the line laser sweeps over the crack defect, a thermal resistance effect occurs, hindering the heat flow from conducting in the direction of the crack. This results in an increase in temperature at the edge of the crack, while the internal temperature of the crack forms a significant gradient difference compared to the surrounding defect-free areas. This temperature discrepancy helps characterize the shape of the crack. This phenomenon can be observed by collecting the surface temperature of the specimen using an infrared camera.

### 2.2. Principle of Image Enhancement by High-Frequency Filtering

When detecting defects, small cracks can be challenging to observe clearly. To address this, it is necessary to suppress the thermal wave background in the infrared image and enhance the defect characteristics through image enhancement techniques [21]. Fourier transform is effective for converting images from the spatial domain to the frequency domain, which aids in analyzing the frequency components. The image can be treated as a two-dimensional signal in the spatial domain, and the Fourier transform decomposes it into various frequency components:

(3) $$F\left(u,v\right)=\sum_{x=0}^{M-1}\sum_{y=0}^{N-1}I\left(x,y\right)e^{-j2\pi \left(\frac{ux}{M}+\frac{vy}{N}\right)}$$

where *I*(*x*, *y*) is the pixel value of the input image in the spatial domain. *f*(*u*, *v*) is the complex representation of the image in the frequency domain, containing the magnitude and phase. *M* and *N* are the width and height of the image. *u* and *v* are the horizontal and vertical coordinates in the frequency domain. *e^-j^*^2*π*^ is the complex exponential function that decomposes the signal into different frequency components.

The low-frequency portion of the Fourier spectrum computed directly by spectral centering is distributed in the corners of the image for easy observation and manipulation:

(4) $$F_{\mathrm{shift}}\left(u,v\right)=F\left(u,v-\frac{N}{2}\right)$$

Subsequently, the energy spectrum is computed in the frequency domain, where the magnitude of the frequency domain represents the intensity of each frequency component in the signal. By squaring the values, the energy spectrum enhances high-frequency components, making weak frequency features more prominent. The magnitude in the frequency domain can be expressed as:

(5) Fu,v=ReFu,v2+ImFPCAu,v2

The frequency energy spectrum is the square of the amplitude in the frequency domain:

(6) $$E\left(u,v\right)={\left|F\left(u,v\right)\right|}^{2}$$

After obtaining the energy spectrum of the image, a Gaussian-type filter is selected for image enhancement in order to enhance the defects and weaken the background:

(7) $$H\left(u,v\right)=\exp\left(-\frac{d{\left(u,v\right)}^{2}}{2\sigma^{2}}\right)$$

where *d*(*u*, *v*) is the distance from the frequency domain point to the center, and *σ* determines the width of the filter and affects the enhancement range. The filtering method can be expressed as:

(8) $$F^{\prime }\left(u,v\right)=F_{\mathrm{shift}}\left(u,v\right)\cdot \left(1-H\left(u,v\right)\right)$$

The enhanced frequency domain image is converted back to the spatial domain to obtain the enhanced image:

(9) $$I^{\prime }\left(x,y\right)=\frac{1}{MN}\sum_{x=0}^{M-1}\sum_{y=0}^{N-1}F^{\prime }\left(u,v\right)e^{-j2\pi \left(\frac{ux}{M}+\frac{vy}{N}\right)}$$

## 3. Experimental Setup

### 3.1. Experimental System

**Figure 1:**
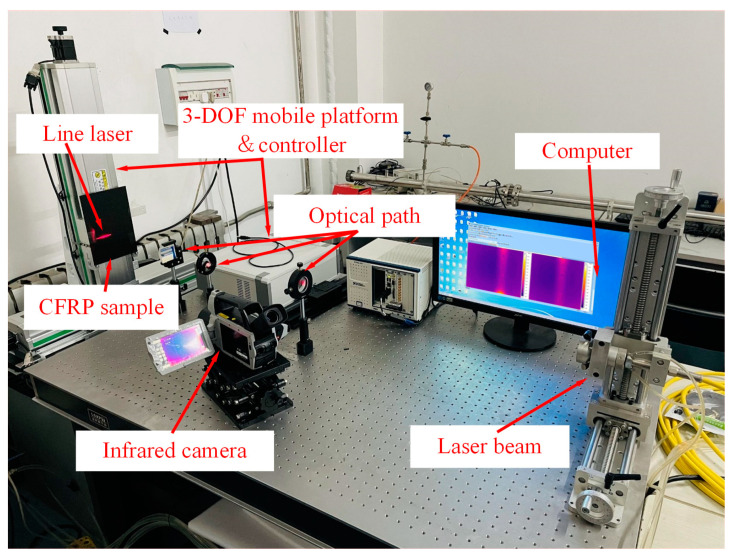
Line laser scanning infrared thermography detection system.

The experimental setup used in this study is shown in Figure 1. A semiconductor laser emitting at a wavelength of 915 nm serves as the excitation source, with a water-cooling device ensuring proper operation by maintaining the laser’s temperature. During the inspection, the CFRP sample is secured on a 3D optical moving platform. As the platform moves, the line laser scans the CFRP sample at a speed of 5 mm/s. Throughout the scanning process, an infrared camera captures infrared image sequences to monitor temperature variations on the surface of the CFRP sample. The thermal imaging camera operates at a frame capture frequency of 10 Hz. Both the infrared camera and the laser source are positioned on the same side of the specimen, implementing a reflective inspection approach.

### 3.2. CFRP Sample

**Figure 2:**
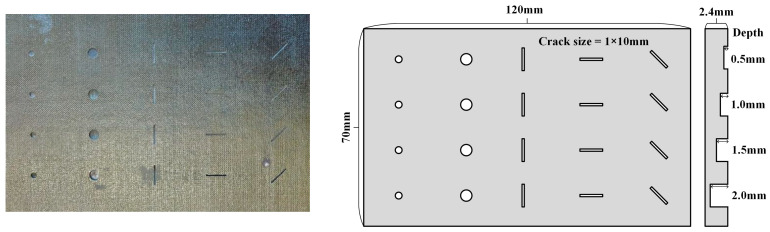
Specimen A: manually layered CFRP multi-angle crack specimen.

**Figure 3:**
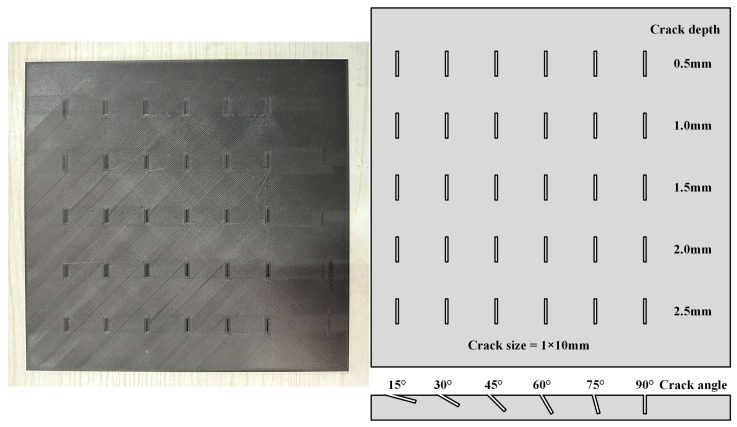
Specimen B: 3D printed CFRP multi-angle opening crack specimen.

**Figure 4:**
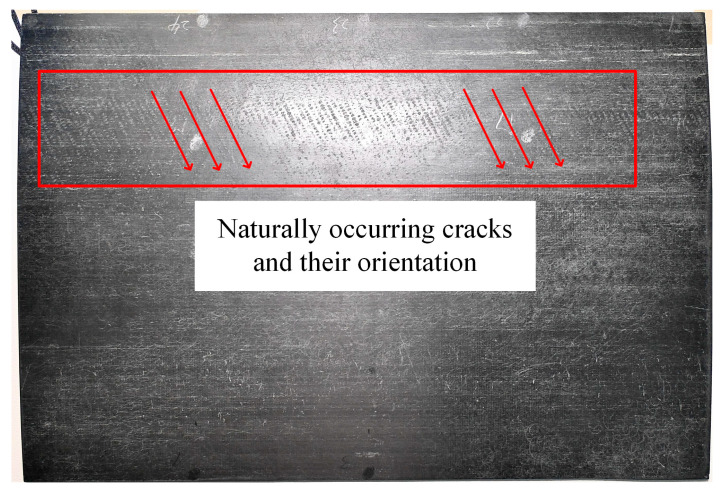
Specimen C: naturally occurring scratches in CFRP deflectors.

This study involved two CFRP specimens: Specimen A, which featured three horizontal angular crack defects with varying angles, and Specimen B, which contained longitudinal angular crack defects. The defects in Specimen A had four distinct depths, with each crack oriented perpendicularly to the specimen’s surface, as schematically illustrated in Figure 2. In Specimen B, the defects exhibited five different depths, with each row of crack openings forming a distinct angle relative to the specimen’s surface, as depicted schematically in Figure 3. Sample B was fabricated using fused deposition modeling (FDM) with a nozzle temperature of 300 °C, a printing speed of 45 mm/s, and a hot bed temperature of 50 °C. After printing, the sample was annealed at 80 °C for 24 h and subsequently dried for 48 h to complete the final molding process. Additionally, we added specimen C. As shown in Figure 4, specimen C is a deflector plate removed from a subway that has been in high-speed operation for an extended period. Its surface exhibits very shallow diagonal scratches, which are naturally occurring cracks. All parameters of the specimens are summarized in Table 1.

## 4. Experimental Results and Analysis

### 4.1. Thermography Results of Line Laser Scanning on Cracks in Specimen A with Different Orientations

**Figure 5:**
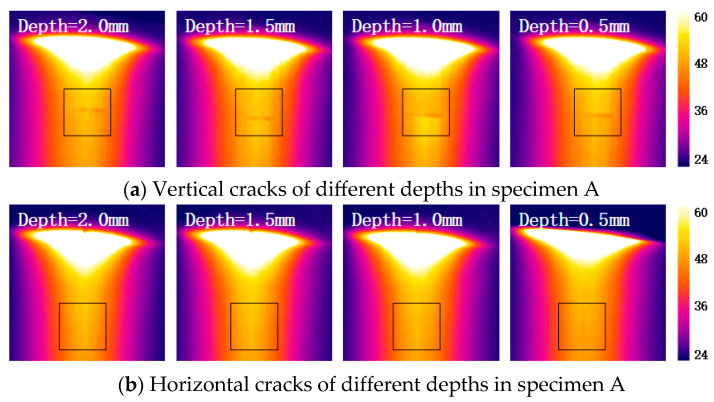

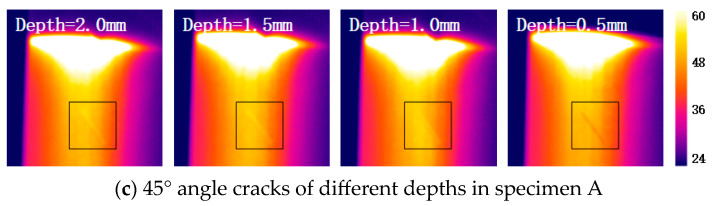
Infrared image of a crack in specimen A swept by a line laser for 1 s.

To investigate the relationship between the line laser scanning direction and the crack orientation, we first examined the cracks in Specimen A. The cracks were exposed to a line laser beam for 1 s. Figure 5 presents the infrared image of Specimen A’s cracks after being scanned by the line laser. The vertical crack is perpendicular to the line laser beam, the horizontal crack is parallel to it, and the diagonal crack forms a 45° angle to the beam.

Although all three types of cracks impede heat transfer during inspection, the mechanisms differ. The vertical crack is continuously excited as the line laser scans over it, causing heat flow to be obstructed on both sides of the crack. This results in higher temperatures at the center of the crack in most cases. For horizontal cracks, the excitation is brief during the scanning process. Heat flow toward the crack is hindered in the defect-free areas on either side, leading to lower temperatures at the crack center compared to the surrounding defect-free regions. Oblique cracks, while also continuously excited, do not receive consistent exposure to the highest line laser power. As a result, the temperature gradient between the crack center and the surrounding defect-free areas is less pronounced [22]. However, it remains true that the deeper the crack, the more pronounced the hindering effect on heat flow conduction, resulting in a greater temperature difference compared to the surrounding areas. As such, shallow cracks are more difficult to detect than deeper cracks [23].

**Figure 6:**
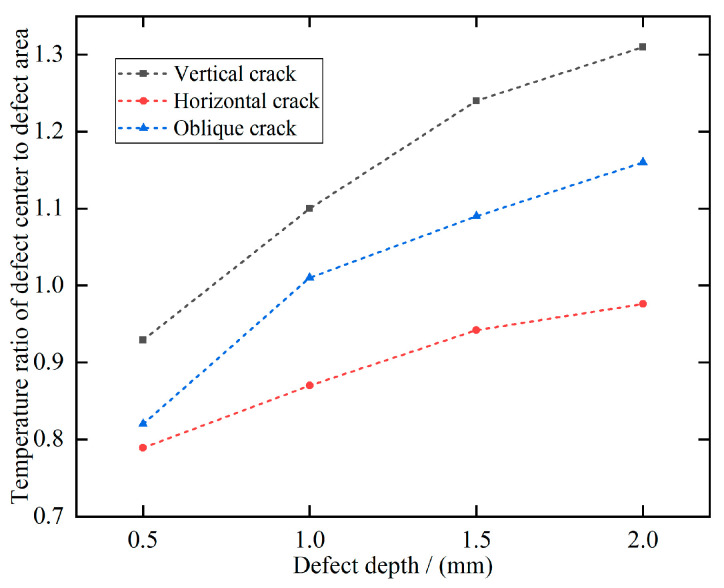
Ratio of crack center to regional mean temperature boxed in Figure 5.

The ratio of the temperature at the center of the crack to the mean temperature of the boxed area was calculated to explore the effect of crack orientation on heat conduction hindrance, referred to as the defect contrast. Figure 6 illustrates the temperature contrast for cracks with varying orientations and depths. The results indicate that deeper defects exhibit higher contrast, making them more prominent in the image, while contrast gradually diminishes as defect depth decreases.

Additionally, vertical cracks generally show higher temperatures compared to the surrounding regions, whereas horizontal cracks tend to exhibit lower temperatures than their surroundings. Oblique cracks display an intermediate behavior. Based on the defect temperature contrast ratio, it is evident that horizontal cracks are the most challenging to detect. However, the factors influencing detection effectiveness are not limited to the scanning direction and crack orientation; the layup direction also plays a critical role in the detection performance of manually layered CFRP.

In addition to the angle of cracks relative to the line laser, the layup direction of manually layered CFRP significantly influences the crack detection effect. In this study, the layup direction of Specimen A was aligned with the line laser scanning direction. As a result, horizontal cracks were consistent with the layup direction, while vertical cracks were perpendicular to it.

Composites typically consist of multiple layers stacked together, and when fibers are aligned in a single direction within the layup, thermal conductivity is primarily governed by the fibers themselves. Numerous studies have shown that in CFRP laminates, thermal conductivity along the fiber direction is significantly higher than that perpendicular to it. As a result, heat conduction is more efficient along the layup direction [24,25]. When cracks align with this direction, their ability to impede heat flow is reduced, leading to minimal temperature differences compared to defect-free regions and producing weak signals in thermal images. Conversely, cracks oriented perpendicular to the layup direction create a more pronounced obstruction to heat transfer, generating significant temperature differences with the surrounding material and resulting in stronger crack signals in thermal images. However, for cross-layered CFRP, the thermal conductivity is nearly uniform across the horizontal plane, which helps to mitigate differences in detection results that would otherwise arise due to variations in thermal conduction.

**Figure 7:**
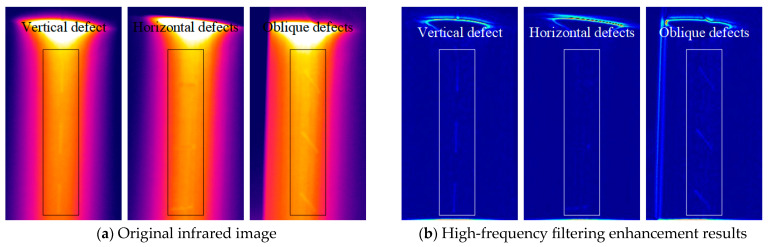
Before and after processing images of three cracks detected by a line laser.

Figure 7 presents pre- and post-processing images of the line laser detection for the three types of cracks. By applying the high-frequency filtering method outlined in Section 2.2, the contours of vertical and oblique cracks appear more distinct due to their stronger signal strengths. Conversely, horizontal cracks exhibit weaker signals, making the defects more challenging to observe clearly.

In summary, for manually layered CFRP, cracks aligned with the layup direction produce weaker signals and poorer detection results. In contrast, cracks at an angle to the layup direction yield stronger signals and superior detection outcomes.

### 4.2. Thermography Results of Line Laser Scanning on Cracks with Different Oblique Angles in Specimen B

In 3D-printed CFRP specimens, the influence of the layup direction on the detection effect is eliminated due to the one-piece molding process. As discussed in Section 4.1 and in much of the current research, the relationship between crack depth and detection effect has been extensively explored, with crack depth commonly used as a key evaluation parameter.

In Specimen B, however, the study focuses on cracks with varying opening angles—an aspect that has been largely overlooked in existing research. Unlike the typical assumption of cracks being perpendicular to the specimen surface, the presence of cracks with different opening angles more accurately represents real-world defect scenarios. This provides a more comprehensive understanding of defect detection and evaluation in practical applications.

**Figure 8:**
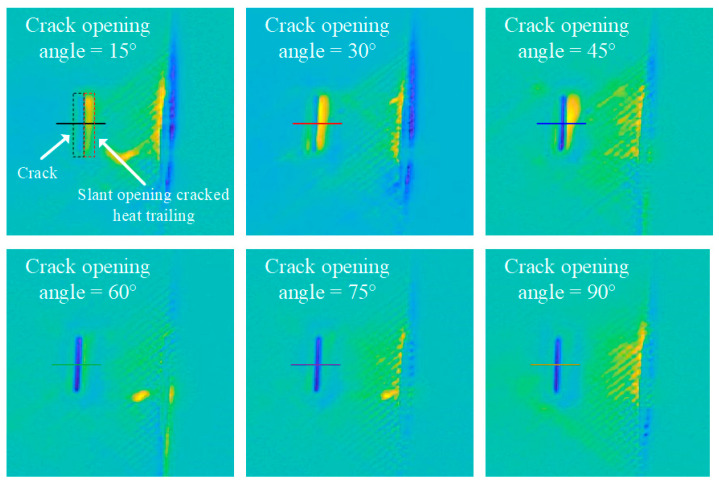
Differential images of cracks with depths of 1.5 mm and different opening angles.

In this study, a line laser was employed to scan and detect a row of cracks with the same depth but varying opening angles. To eliminate the thermal drag caused by the line laser scanning, differential images were calculated by selecting a reference image. As shown in Figure 8, the differential images of defects with a depth of 1.5 mm and different crack opening angles are presented.

**Figure 9:**
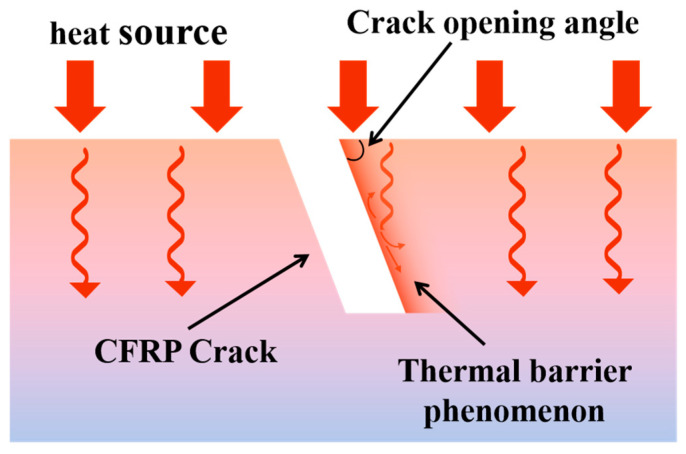
Schematic illustration of the phenomenon of obstruction of heat conduction by oblique opening crack.

It can be observed that cracks with different opening angles exhibit varying degrees of thermal drag. Smaller opening angles result in more pronounced thermal trailing; however, at the same time, the central region of these cracks has a weaker effect on obstructing heat flow, leading to lower contrast in thermal imaging. This occurs because cracks with an opening angle disrupt heat conduction along their direction, producing a thermal trailing effect in the crack opening direction [26]. As shown in Figure 9, heat conduction above an angled opening crack is significantly impeded, resulting in a distinct thermal wake in the surface temperature distribution captured by thermal imaging.

**Figure 10:**
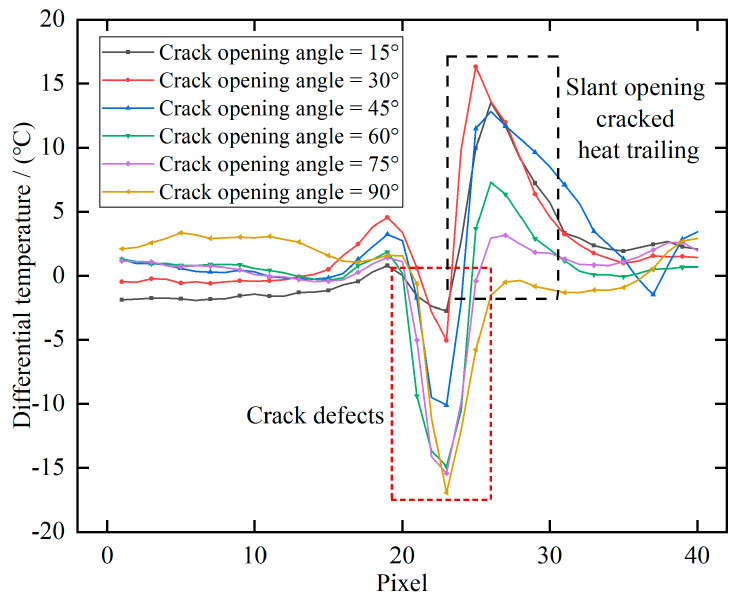
Line temperature response of the crack in Figure 8.

Figure 10 illustrates the line temperature response corresponding to Figure 8. It is evident that as the crack opening angle increases, the extent of the thermal drag tail diminishes, and the associated temperature decreases. Concurrently, larger crack opening angles correspond to lower central crack temperatures and exhibit a stronger hindering effect on heat transfer.

**Figure 11:**
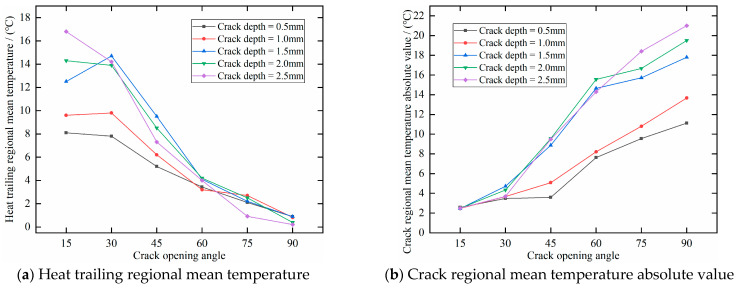
Trend of regional temperature with crack opening angle.

Through the above analysis, the image of a crack with an opening angle that is detected can be divided into a crack region and a crack heat trailing region, as shown in Figure 8. The opening angle of the crack can be determined by comparing the temperature data from these two regions. Since the temperature in the crack region is typically negative and the temperature in the crack heat trailing region is positive in the differential image, the difference caused by the crack opening angle can be expressed using the ratio of the absolute values of the temperatures in the two regions, which is referred to as the crack absolute temperature difference ratio (ADTR). The formula can be expressed as:

(10) $$ADTR=\frac{\left|T_{\text{average-tailing}}\right|}{\left|T_{\mathrm{average-crack}}\right|}$$

where *T*_average-crack_ denotes the differential mean temperature in the crack region, and *T*_average-tailing_ denotes the differential mean temperature in the crack heat-trailing region. Figure 11 illustrates the temperature trends in two regions relative to the crack opening angle. Figure 11a depicts the differential temperature trend in the heat trailing region, while Figure 11b shows the trend of the absolute differential temperature in the cracked region. It can be observed that as the crack opening angle increases, the average temperature in the heat trailing region decreases, while the absolute average temperature in the cracked region increases. This trend remains consistent across the different crack depths. However, from the curves in Figure 11, it is difficult to clearly fit the temperature trends for both regions. Therefore, it is necessary to investigate the fitting relationship of the ratio of the absolute temperature difference in the cracks to better understand the underlying pattern.

**Figure 12:**
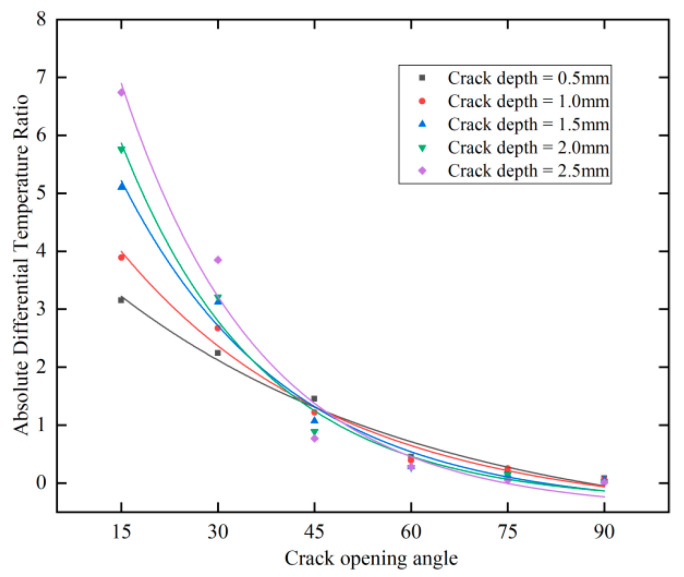
Fitted curves of crack absolute temperature difference ratio versus crack opening angle for cracks of different depths.

Figure 12 shows the fitted curves of the crack absolute temperature difference ratio versus crack opening angle for cracks of different depths. From the figure, it can be observed that as the crack opening angle increases, the crack absolute temperature difference ratio decreases. In other words, a larger crack opening angle results in a stronger hindering effect of the crack center on the temperature and a weaker thermal towing phenomenon. Additionally, it is evident that for cracks with smaller opening angles, the deeper the crack, the higher the absolute temperature difference ratio. Conversely, for cracks with larger opening angles, the absolute temperature difference ratio decreases with greater crack depth. This phenomenon suggests that when the crack opening angle is small (less than 60°), the absolute temperature difference ratio is more sensitive to variations in the opening angle. However, when the opening angle exceeds 60°, the absolute temperature difference ratio remains nearly unchanged. Regardless of the opening angle, deeper cracks consistently exhibit a stronger obstruction to heat transfer, highlighting crack depth as a key factor in determining the extent of heat conduction blockage.

Overall, the fitted curves for the crack absolute temperature difference ratio versus crack opening angle follow an exponential trend. For the performance metrics of linear regression models, we typically use *R*^2^ (R-square) and mean square error (MSE) to evaluate the model’s fit and prediction error [27]. The *R*^2^ value measures the proportion of the variance in the dependent variable that is explained by the model. It is calculated using the following formula:

(11) $$R^{2}=1-\frac{SS_{residual}}{SS_{total}}$$

where *SS_residual_* is the residual sum of squares, which represents the sum of squares of the residuals of the model fit. *SS_total_* is the total sum of squares, which represents the sum of squares of the difference between the dependent variable and its mean. *R*^2^ takes values between 0 and 1, with the closer to 1 indicating the better the model fits the data.

*MSE* is the average of the squares of the differences between the actual observations and the model predictions. It is calculated by the formula:

(12) $$MSE=\frac{1}{n}{\sum_{i=1}^{n}\left(y-{\hat{y}}_{i}\right)}^{2}$$

where *n* is the number of samples. *y_i_* is the *i*th observation. ${\hat{y}}_{i}$ is the corresponding model prediction. A smaller *MSE* means that the model fits the data better.

Table 2 presents the fitting result functions and metrics shown in Figure 12. All the fitting functions are first-order exponential decay functions, where A_1_ represents the initial amplitude, t_1_ is the time constant that controls the rate of decay, and y_0_ is a constant term indicating the horizontal offset. The results demonstrate that the average R² for fitting the absolute temperature difference ratio to the crack opening angle for cracks of various depths is 0.9828, and the average MSE is 0.1287, validating the conclusions of this study.

### 4.3. Thermography Results of Line Laser Scanning on Naturally Occurring Scratches in Specimen C

**Figure 13:**
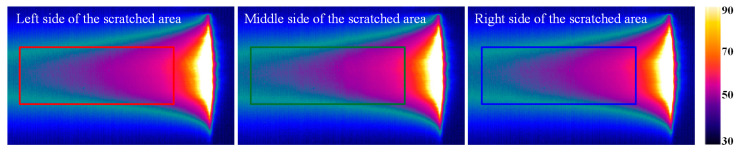
Raw infrared images of natural scratches detected by line laser scanning.

**Figure 14:**
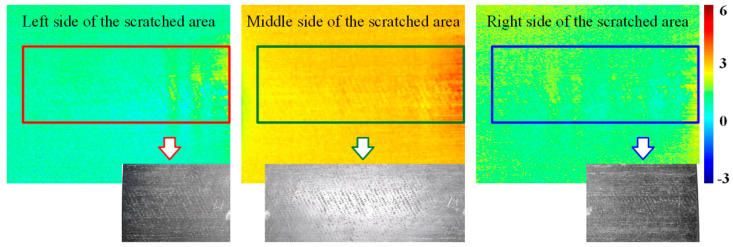
High-frequency filtering results for differential images of natural scratches.

To validate the effectiveness of this study in detecting naturally occurring cracks, specimen C was examined using line laser scanning. As shown in the physical image of specimen C, the depth of the scratches is very shallow, with the central area being more pronounced compared to the two sides, which are nearly unnoticeable under dark illumination. Figure 13 presents the original images of the line laser scanning across the left, center, and right sides of the scratched area. From these images, it is clear that the trailing of the line laser makes it difficult to discern the exact shape of the scratches. To overcome this, a reference image was selected, and the differential image for detecting the scratches was calculated. The differential image was then processed using the high-frequency filtering method outlined in Section 2.2, with the results shown in Figure 14.

It can be observed that the central scratched region, where the scratches are more severe, contains more high-frequency components, leading to a higher overall magnitude in the image. In contrast, the two sides of the scratched area, where the scratches are less pronounced, exhibit fewer high-frequency components and are less visually distinct.

Overall, the method proposed in this study effectively detects naturally occurring microcracks (scratches) and extracts their features through a series of processing steps, confirming the feasibility and applicability of the approach.

## 5. Conclusions

In this study, line laser scanning infrared thermography was employed to detect cracks in both manually layered CFRP and 3D-printed CFRP. For manually layered CFRP, cracks were introduced in three orientations: parallel, perpendicular, and 45° to the layup direction. The thermal imaging results revealed that when the line laser beam was parallel to the crack direction, the display contrast was lower than in the other two orientations. High-frequency filtering enhancement indicated that cracks aligned with the layup direction did not significantly hinder thermal conduction due to the fiber-dominated heat transfer mechanism. Since the thermal conductivity along the fiber direction is higher, cracks had a weaker obstructing effect on heat flow, resulting in lower contrast in the frequency-energy signal.

For 3D-printed CFRP, where the layup direction effect is eliminated, six types of cracks with different opening angles and five depth levels were analyzed. Differential infrared images showed that cracks with smaller opening angles exhibited more pronounced thermal trailing while simultaneously providing weaker obstruction to heat flow at their center, leading to lower contrast. This phenomenon occurs because cracks with an opening angle primarily hinder heat conduction along the crack direction, inducing thermal drag in the opening direction. To quantify the relationship between crack opening angle and heat conduction obstruction, the crack images were divided into two regions: the crack region and the thermal trailing region, and the absolute temperature difference ratio between these two regions was computed. The results indicated that when the crack opening angle is less than 60°, the absolute temperature difference ratio is highly sensitive to changes in the opening angle, whereas when the angle exceeds 60°, the ratio remains nearly unaffected. The fitting results yielded an average *R*^2^ of 0.9828 and an MSE of 0.1287, confirming the validity of the proposed methodology.

Finally, CFRP deflector specimens with naturally occurring surface microcracks (scratches) were examined. Through high-frequency filtering of their differential images, the features of the scratches, which were nearly invisible under low light conditions, were effectively extracted. This successfully demonstrates the generalizability of the method proposed in this study.

This study on the detection of diagonal opening cracks in CFRP provides valuable insights for the quality assessment of 3D-printed CFRP. In the future, this technique can be further extended to the inspection of complex structures with intricate crack defects. Additionally, integrating intelligent data processing and automated detection algorithms could enhance detection efficiency and broaden its applicability.

## Figures and Tables

**Table 1:** Parameters of CFRP samples.

Parameter Name	Value
Specimen A type	T800
Specimen A crack horizontal angle	0°/45°/90°
Specimen A size (L × W × H) (mm)	70 × 120 × 2.4
Specimen A molding method	Manually layered 0° unidirectional
Specimen B type	PolyMide™ PA6-CF
Specimen B crack opening angle	15°/30°/45°/60°/75°
Specimen B size (L × W × H) (mm)	300 × 300 × 3
Specimen B molding method	3D printing
Crack size (L × W) (mm)	10 × 1
Specimen C type	T300
Specimen C size (L × W × H) (mm)	300 × 200 × 2.7

**Table 2:** Functions and metrics for fitting.

Crack Depth (mm)	0.5	1.0	1.5	2.0	2.5
Function	y = A_1_ × exp(−x/t_1_) + y_0_				
y_0_	−0.95	−0.56	−0.44	−0.34	−0.47
A_1_	5.67	7.09	10.15	12.27	14.71
t_1_	48.98	33.90	25.68	21.99	21.67
Mean square error	0.0426	0.0652	0.1145	0.1254	0.2957
R-square	0.9834	0.9837	0.9844	0.9859	0.9767

## Data Availability

Data are contained within the article.
